# High-Resolution Esophageal Manometry in Mongolia: The First National Experience and Diagnostic Spectrum of Esophageal Motility Disorders

**DOI:** 10.3390/jcm15197420

**Published:** 2026-09-24

**Authors:** Amarjargal Batdelger, Oyuntugs Byambasukh, Bayarmaa Nyamaa, Sarantuya Gidaagaya

**Affiliations:** 1 Department of Gastroenterology, Mongolian National University of Medical Sciences, Ulaanbaatar 14210, Mongolia; amd23f006@gt.mnums.edu.mn or amarjargal.b@intermed.mn (A.B.); bayarmaa.n@mnums.edu.mn (B.N.); 2 Department of Gastroenterology and Endoscopy, Intermed IUHW Hospital, Ulaanbaatar 17040, Mongolia; 3 Department of Endocrinology, Mongolian National University of Medical Sciences, Ulaanbaatar 14210, Mongolia

**Keywords:** high-resolution esophageal manometry, esophageal motility disorders, esophagogastric junction outflow obstruction, gastroesophageal reflux disease

## Abstract

**Background:** High-resolution esophageal manometry (HREM) is the reference standard for evaluating esophageal motility disorders; however, data from low- and middle-income countries remain limited. As HREM has only recently become available in Mongolia, this study aimed to provide the first comprehensive characterization of HREM in Mongolia by characterizing referral patterns, the diagnostic spectrum of esophageal motility disorders, and clinical factors associated with major motility disorders. **Methods:** We conducted a retrospective observational study of 998 consecutive adults who underwent HREM at Intermed IUHW Hospital, the only center currently providing routine clinical HREM services in Mongolia (*n* = 998). Manometric findings were interpreted according to the Chicago Classification version 4.0. Multivariable logistic regression was performed as an exploratory analysis to identify clinical factors independently associated with major esophageal motility disorders. A sensitivity analysis was performed after excluding patients with a manometric esophagogastric junction outflow obstruction (EGJOO) pattern. **Results:** Overall, 696 (69.7%) patients were referred for evaluation of suspected gastroesophageal reflux disease or for preoperative anti-reflux assessment, whereas 302 (30.3%) were referred because of suspected esophageal motility disorders. Normal esophageal motility was identified in 61.9% of patients, while ineffective esophageal motility was the most common abnormal finding (26.9%). Major esophageal motility abnormalities were identified in 11.2% of patients, most commonly a manometric EGJOO pattern (6.4%) and achalasia (2.7%). Increasing age (adjusted odds ratio [aOR] 1.35 per 10-year increase, 95% CI 1.12–1.61), lower body mass index (aOR 0.90 per kg/m^2^, 95% CI 0.85–0.95), dysphagia (aOR 1.95, 95% CI 1.20–3.15), and referral for suspected motility disorders (aOR 5.47, 95% CI 3.22–9.31) were independently associated with major esophageal motility abnormalities. The exploratory model demonstrated moderate discrimination (AUC 0.791, 95% CI 0.741–0.840). **Conclusions:** Most patients undergoing HREM demonstrated either normal esophageal motility or ineffective esophageal motility, whereas major motility abnormalities were relatively uncommon. Older age, lower body mass index, dysphagia, and referral for suspected motility disorders were independently associated with major motility abnormalities. These findings provide baseline data on the diagnostic spectrum of HREM among referred patients in Mongolia and may inform future prospective studies of HREM referral strategies.

## 1. Introduction

Esophageal motility disorders comprise a heterogeneous group of conditions characterized by impaired esophageal peristalsis and/or abnormal lower esophageal sphincter function, resulting in symptoms such as dysphagia, non-cardiac chest pain, regurgitation, and reflux-related complaints. These disorders range from minor abnormalities with uncertain clinical significance to major motility disorders, including achalasia, esophagogastric junction outflow obstruction (EGJOO), distal esophageal spasm, hypercontractile esophagus, and absent contractility, which often require disease-specific management. Dysphagia is one of the most common presenting symptoms, affecting approximately 16% of adults worldwide and substantially impairing quality of life and healthcare utilization [1,2].

High-resolution esophageal manometry (HREM) has transformed the evaluation of esophageal motor function by providing detailed pressure topography of the esophagus and esophagogastric junction. Compared with conventional manometry, HREM offers improved spatial resolution, standardized pressure metrics, and more consistent diagnostic interpretation, making it the reference method for evaluating suspected esophageal motility disorders and an essential investigation before anti-reflux surgery and in patients with unexplained dysphagia or non-cardiac chest pain [3,4,5]. The introduction of the Chicago Classification version 4.0 has further standardized the interpretation of HREM findings by emphasizing clinically relevant diagnoses, standardized testing protocols, upright swallows, provocative maneuvers, and integration of manometric findings with patient symptoms and supportive investigations when appropriate [6,7,8,9].

Importantly, the Chicago Classification version 4.0 distinguishes a manometric EGJOO pattern from a clinically relevant or conclusive diagnosis of EGJOO, which requires clinically relevant symptoms and supportive evidence of esophagogastric junction obstruction, such as abnormal findings on timed barium esophagogram and/or functional lumen imaging probe (FLIP) testing [6,10,11].

Studies from Europe, North America, and East Asia have shown that normal esophageal motility and ineffective esophageal motility are the most common HREM findings, whereas major motility disorders account for only a minority of examinations [8,9,12]. HREM also plays an important role in evaluating patients with gastroesophageal reflux disease (GERD), particularly before anti-reflux surgery, and several manometric parameters have been associated with esophageal acid exposure, reflux burden, and postoperative outcomes, supporting its role as an essential component of pre-intervention physiologic assessment in current international guidelines and consensus recommendations [12,13,14,15,16,17,18,19].

Consequently, understanding referral patterns and the distribution of HREM diagnoses is essential for interpreting HREM findings in routine clinical practice and informing future studies of appropriate referral strategies.

Despite increasing adoption of HREM worldwide, evidence from low- and middle-income countries remains limited, and no published study has comprehensively described HREM findings in Mongolia. HREM has only recently been introduced into routine clinical practice in the country, with Intermed IUHW Hospital currently providing the only clinical HREM service. Consequently, the diagnostic spectrum, referral patterns, and clinical characteristics of patients undergoing HREM in Mongolia remain incompletely characterized.

Therefore, this study aimed to describe the first comprehensive experience with HREM in Mongolia by characterizing referral patterns, the diagnostic spectrum of esophageal motility disorders according to the Chicago Classification version 4.0, and the clinical factors independently associated with major esophageal motility disorders among adults undergoing HREM in routine clinical practice.

## 2. Materials and Methods

### 2.1. Study Design and Population

We conducted a retrospective observational study of consecutive adult patients who underwent high-resolution esophageal manometry (HREM) at Intermed IUHW Hospital, Ulaanbaatar, Mongolia. Intermed IUHW Hospital is a tertiary referral hospital and currently the only medical center providing routine clinical HREM services in Mongolia. Patients are referred from gastroenterology clinics and hospitals throughout the country for evaluation of esophageal symptoms and preoperative assessment before anti-reflux surgery.

All HREM examinations performed between September 2022 and March 2026 were screened for eligibility. Adults aged ≥ 18 years who underwent clinically indicated HREM were eligible for inclusion. Indications included referral for evaluation of suspected gastroesophageal reflux disease (GERD), preoperative anti-reflux assessment, dysphagia, non-cardiac chest pain, regurgitation, and suspected esophageal motility disorders. Patients with previous upper gastrointestinal surgery, prior peroral endoscopic myotomy, technically inadequate HREM studies, incomplete clinical information, or unresolved data-quality inconsistencies were excluded. After applying the eligibility criteria and quality-control procedures, 998 patients were included in the final analysis (Figure 1).

The study was approved by the Medical Ethics Committee of the Mongolian National University of Medical Sciences (IRB No. 2024/3-07) and was conducted in accordance with the principles of the Declaration of Helsinki. Given the retrospective nature of the study and the use of anonymized routinely collected clinical data, the requirement for informed consent was waived.

### 2.2. High-Resolution Esophageal Manometry Procedure

All HREM examinations were performed according to standardized clinical protocols by trained gastroenterologists using a solid-state high-resolution impedance manometry system (Diversatek, Milwaukee, WI, USA), following the ACG Clinical Guideline for Esophageal Physiologic Testing, the Chicago Classification version 4.0, and its accompanying technical review [3,5,6]. Patients fasted for at least 6 h before the examination. Following transnasal catheter placement, a baseline recording was obtained. The standardized protocol included ten 5 mL water swallows in the supine position, followed by 5 mL water swallows in the upright position. Provocative maneuvers included multiple rapid swallows (MRS; five consecutive 2 mL swallows) and a rapid drink challenge (RDC; 100 mL water ingested as rapidly as tolerated), performed according to the institutional protocol [4,6,7]. The EGJ complex was evaluated during quiet respiration using a swallow-free and artifact-free segment of the baseline recording. In accordance with the technical recommendations of the Chicago Classification version 4.0, manometric EGJ morphology was determined based on the spatial relationship among the lower esophageal sphincter (LES), crural diaphragm (CD), and respiratory inversion point (RIP). EGJ morphology was classified as type I when the CD was superimposed on the LES and the RIP was located proximal to the EGJ complex; type II when LES–CD separation was present and the RIP was located proximal to the CD; and type III when LES–CD separation was present and the RIP was located proximal to the LES [6,7,8]. LES length was measured as the axial length of the lower esophageal sphincter high-pressure zone and was reported in centimeters. Contractile reserve was assessed using the DCI ratio, calculated as the post-MRS DCI divided by the mean DCI of the preceding single wet swallows. A DCI ratio greater than 1.0 was considered indicative of preserved contractile reserve. Complete bolus transit was assessed using concurrent impedance recordings and was defined as complete bolus passage from the proximal to the distal impedance segments. It was reported as the percentage of evaluated swallows demonstrating complete bolus transit [15,20].

Manometric studies were interpreted by experienced physicians according to the diagnostic criteria of the Chicago Classification version 4.0 [5]. Diagnoses included normal esophageal motility, ineffective esophageal motility (IEM), absent contractility, achalasia, a manometric EGJOO pattern, distal esophageal spasm, hypercontractile esophagus, and other esophageal motility disorders where appropriate. In this study, EGJOO was recorded as a manometric pattern based on the HREM findings and was not considered a definitive clinical diagnosis in the absence of systematically available supportive investigations.

### 2.3. Data Collection and Study Variables

Clinical information was extracted retrospectively from the hospital electronic medical record system and the HREM reporting database. Demographic variables included age, sex, height, weight, and body mass index (BMI). Presenting symptoms included dysphagia, heartburn, regurgitation, non-cardiac chest pain, globus sensation, chronic cough, and other upper gastrointestinal symptoms recorded at the time of referral.

Patients were categorized according to referral indication into two groups: (1) evaluation of suspected GERD or preoperative assessment before anti-reflux surgery and (2) suspected esophageal motility disorders. HREM variables extracted from the reports included integrated relaxation pressure (IRP), distal contractile integral (DCI), lower esophageal sphincter (LES) basal pressure, DCI ratio, complete bolus transit, and multiple rapid swallow (MRS) responses, when available.

### 2.4. Outcome Definitions

The primary outcome was the presence of a major esophageal motility disorder, defined according to the Chicago Classification version 4.0. Major motility disorders included achalasia, a manometric EGJOO pattern, distal esophageal spasm, hypercontractile esophagus, and absent contractility. Patients with normal esophageal motility or minor motility disorders, including ineffective esophageal motility, were classified as the comparison group.

For the sensitivity analysis, the outcome was redefined by excluding patients with a manometric EGJOO pattern from the major motility disorder group, as the clinical significance of this pattern may vary according to supportive investigations and clinical context.

### 2.5. Statistical Analysis

Continuous variables were summarized as mean ± standard deviation (SD) for normally distributed variables or median with interquartile range (IQR) for non-normally distributed variables. Categorical variables were expressed as frequencies and percentages. Comparisons between referral groups and between patients with and without major esophageal motility disorders were performed using the independent-samples t-test or Mann–Whitney U test for continuous variables, as appropriate, and Pearson’s chi-square test or Fisher’s exact test for categorical variables.

Candidate variables were selected based on clinical relevance and findings from univariable analyses. The final multivariable logistic regression model included age, sex, body mass index, dysphagia, heartburn, and referral indication, with all variables entered simultaneously. The model was used to identify clinical factors independently associated with major manometric motility abnormalities. Results were reported as adjusted odds ratios (aORs) with 95% confidence intervals (CIs). Multicollinearity among the included predictors was assessed using tolerance and variance inflation factors (VIFs); no evidence of problematic multicollinearity was observed (tolerance, 0.654–0.932; VIF, 1.073–1.528). The multivariable analysis was performed using complete cases, with no imputation of missing data.

Model discrimination was assessed using receiver operating characteristic (ROC) curve analysis and the area under the ROC curve (AUC). Model calibration was assessed using the Hosmer–Lemeshow goodness-of-fit test. The multivariable analysis was considered exploratory, and the model was not intended to serve as a clinical prediction or screening tool. A sensitivity analysis was conducted using the alternative outcome definition described above.

All statistical analyses were conducted using IBM SPSS Statistics version 31.0 (IBM Corp., Armonk, NY, USA). Two-sided *p* values < 0.05 were considered statistically significant.

## 3. Results

### 3.1. Study Population and Diagnostic Spectrum

A total of 1033 HREM examinations were screened for eligibility. After application of the predefined eligibility criteria and data-quality review, 998 consecutive adult patients were included in the final analysis (Figure 1).

Among the included patients, 696 (69.7%) were referred for evaluation of suspected gastroesophageal reflux disease (GERD) or for preoperative anti-reflux assessment, whereas 302 (30.3%) were referred for suspected esophageal motility disorders. Patients referred for suspected motility disorders were younger, had a lower body mass index, and more frequently presented with dysphagia and non-cardiac chest pain, whereas heartburn, regurgitation, and cough were more common among those referred for suspected GERD evaluation or preoperative anti-reflux assessment. Baseline clinical characteristics according to referral indication are summarized in Table 1.

According to the Chicago Classification version 4.0, normal esophageal motility was identified in 618 patients (61.9%), whereas ineffective esophageal motility was the most common abnormal finding, affecting 268 patients (26.9%). Major esophageal motility disorders were diagnosed in 112 patients (11.2%), including a manometric esophagogastric junction outflow obstruction pattern (6.4%), achalasia (2.7%), absent contractility (1.6%), distal esophageal spasm (0.3%), and hypercontractile esophagus (0.2%). The distribution of HREM diagnoses is shown in Figure 2.

Manometric EGJ morphology was available for all 998 patients. Type I, type II, and type III morphology were identified in 55 (5.5%), 430 (43.1%), and 513 (51.4%) patients, respectively.

### 3.2. High-Resolution Esophageal Manometry Findings According to Referral Indication

Patients referred for suspected esophageal motility disorders demonstrated significantly higher integrated relaxation pressure, distal contractile integral, lower esophageal sphincter pressure, and post-multiple rapid swallow distal contractile integral than those referred for evaluation of suspected GERD or for preoperative anti-reflux assessment (all *p* < 0.01). Conversely, total lower esophageal sphincter length was significantly shorter in the suspected motility disorder group. The distal contractile integral ratio and complete bolus transit did not differ significantly between the referral groups (Table 2).

### 3.3. Characteristics Associated with Major Esophageal Motility Disorders

Compared with patients with normal or minor motility disorders, those with major disorders had significantly lower body mass index (24.7 ± 4.9 vs. 27.5 ± 4.2 kg/m^2^, *p* < 0.001). Dysphagia was substantially more common among patients with major disorders (50.5% vs. 21.3%, *p* < 0.001), whereas heartburn and regurgitation were less frequent. Referral for suspected motility disorders was strongly associated with major esophageal motility disorders, with 70.5% of affected patients originating from this referral group (Table 3).

### 3.4. Factors Associated with Major Manometric Motility Abnormalities

In univariable logistic regression analyses, lower body mass index, dysphagia, absence of heartburn, absence of regurgitation, and referral for suspected motility disorders were significantly associated with major manometric motility abnormalities. After multivariable adjustment, increasing age (adjusted OR 1.35 per 10-year increase, 95% CI 1.12–1.61), lower body mass index (adjusted OR 0.90 per kg/m^2^, 95% CI 0.85–0.95), dysphagia (adjusted OR 1.95, 95% CI 1.20–3.15), and referral for suspected motility disorders (adjusted OR 5.47, 95% CI 3.22–9.31) remained independently associated with major manometric motility abnormalities, whereas male sex and heartburn were not independently associated with the outcome. Of the 998 participants, 991 (99.3%) had complete data for all variables included in the final multivariable model (Table 4).

### 3.5. Exploratory Assessment of the Multivariable Logistic Regression Model

The multivariable logistic regression model demonstrated moderate discrimination, with an area under the receiver operating characteristic (ROC) curve of 0.791 (95% CI, 0.741–0.840) (Figure 3). The model accounted for 23.6% of the variation in the outcome (Nagelkerke R^2^ = 0.236). At the evaluated classification threshold, sensitivity was 9.9%, and specificity was 99.3%, with an overall classification accuracy of 89.3%. Given the relatively low prevalence of major manometric motility abnormalities, the high overall accuracy was largely influenced by correct classification of patients without the outcome and should not be interpreted as evidence of good screening performance. The Hosmer–Lemeshow goodness-of-fit test indicated suboptimal calibration (*p* = 0.037). These performance measures are presented descriptively because the model was exploratory and was not developed as a clinical prediction or screening tool.

### 3.6. Sensitivity Analysis

Excluding patients with a manometric EGJOO pattern from the definition of major esophageal motility disorders yielded similar findings. Older age, male sex, lower body mass index, dysphagia, and referral for suspected motility disorders remained independently associated with major motility disorders, whereas heartburn was not independently associated with the outcome. The sensitivity analysis showed similar discrimination with AUC of 0.780 (95% CI, 0.712–0.848) (Table 5).

## 4. Discussion

This study represents the first comprehensive description of high-resolution esophageal manometry (HREM) findings in Mongolia and provides important baseline evidence regarding referral patterns, the diagnostic spectrum of esophageal motility disorders, and clinical factors associated with major motility disorders. Four principal findings emerged. First, most patients referred for HREM demonstrated either normal esophageal motility or ineffective esophageal motility, whereas major esophageal motility disorders were identified in only approximately one-tenth of examinations. Second, patients referred for suspected esophageal motility disorders exhibited significantly different manometric characteristics from those referred for evaluation of suspected gastroesophageal reflux disease (GERD) or for preoperative anti-reflux assessment. Third, older age, lower body mass index (BMI), dysphagia, and referral for suspected motility disorders were independently associated with major esophageal motility disorders. Finally, the multivariable logistic regression model showed moderate discrimination (AUC 0.791), but its low sensitivity and suboptimal calibration limit its clinical utility as a prediction or screening tool.

The overall distribution of HREM findings observed in this study is consistent with previous reports from tertiary referral centers. Normal esophageal motility remained the most frequent finding, followed by ineffective esophageal motility (IEM), whereas major motility disorders were comparatively uncommon. Similar diagnostic distributions have been reported in large international HREM cohorts, where normal studies and IEM typically account for most examinations, while achalasia, manometric EGJOO pattern, distal esophageal spasm, and hypercontractile esophagus collectively represent a substantially smaller proportion of referrals [20,21]. In our cohort, a manometric EGJOO pattern was identified considerably more often among patients referred because of suspected esophageal motility disorders than among those referred for GERD evaluation. This finding is consistent with the heterogeneous nature of manometric EGJOO, which requires careful evaluation to distinguish clinically relevant EGJOO from secondary or mechanical causes before therapeutic intervention [6,10,11].

More recently, Mari et al. confirmed that HREM findings differ significantly according to the clinical indication for testing, with the highest diagnostic yield for major motility disorders observed among patients evaluated for dysphagia and recurrent regurgitation/vomiting, supporting the indication-based differences observed in our cohort [12]. These findings suggest that despite differences in healthcare systems and patient populations, the spectrum of esophageal motility disorders encountered in routine clinical practice in Mongolia is broadly comparable to that reported in international HREM cohorts.

The majority of patients referred for GERD evaluation or preoperative assessment before anti-reflux surgery in our cohort likely reflect the increasing use of HREM as part of routine esophageal physiologic testing before anti-reflux interventions. Current international guidelines and consensus statements recommend high-resolution manometry to exclude major esophageal motility disorders, characterize esophageal motor function, and complement reflux monitoring when evaluating patients for anti-reflux intervention [4,19]. These findings support an individualized approach to patient selection for esophageal physiologic testing, which is consistent with the recent AGA Clinical Practice Update recommending tailored diagnostic pathways based on symptom profile and clinical context [21]. Therefore, the high proportion of referrals related to GERD evaluation or anti-reflux surgery observed in our study is consistent with contemporary clinical practice and highlights the expanding role of HREM in the comprehensive physiologic assessment of patients being considered for anti-reflux intervention. The ICARUS consensus further recommends that esophageal manometry should be performed routinely before anti-reflux intervention to exclude major motility disorders, while ambulatory reflux monitoring is recommended when endoscopy does not demonstrate unequivocal reflux esophagitis [16,22].

The predominance of IEM is also clinically relevant. IEM has become increasingly recognized as the most common motility disorder identified during HREM and is frequently encountered among patients with GERD. Previous studies have demonstrated associations between ineffective esophageal clearance, increased esophageal acid exposure, and impaired reflux clearance, although the clinical significance of isolated IEM remains variable [13,14,17]. Because a substantial proportion of patients in our cohort were referred for GERD evaluation or preoperative assessment before invasive anti-reflux therapy, the high prevalence of IEM may reflect the clinical context of the referred population, although a direct association with GERD cannot be established from our data. This interpretation is further supported by the study of Rengarajan et al., who demonstrated that high-resolution manometry of both the esophagogastric junction and esophageal body was associated with esophageal acid burden, highlighting the potential role of HREM as a physiologic tool in the assessment of GERD beyond the diagnosis of primary motility disorders [17].

Patients referred because of suspected esophageal motility disorders demonstrated significantly higher integrated relaxation pressure (IRP), distal contractile integral (DCI), lower esophageal sphincter pressure, and multiple rapid swallow (MRS) distal contractile integral than patients referred for GERD evaluation. These findings are physiologically plausible because disorders such as achalasia and manometric EGJOO pattern are characterized by impaired esophagogastric junction relaxation and altered esophageal contractility, leading to elevated IRP and abnormal pressure metrics. Likewise, MRS responses provide information regarding peristaltic reserve and may help identify impaired esophageal contractile function that is not apparent during single swallows [7,15]. Although MRS DCI was significantly higher in patients with suspected motility disorders, the DCI ratio did not differ significantly between the two groups, suggesting that the relative augmentation of esophageal contractility during multiple rapid swallows was similar despite differences in baseline esophageal motor function. The observed differences are consistent with the clinical indication-based differences in HREM findings observed in our cohort and with previous studies demonstrating a substantially higher diagnostic yield among patients referred for dysphagia than for reflux-related symptoms [7,12,15].

An important finding of this study is that increasing age, lower BMI, dysphagia, and referral for suspected motility disorders were independently associated with major esophageal motility disorders. Dysphagia is the cardinal symptom of achalasia and other clinically significant motility disorders and has consistently been associated with abnormal HREM findings in previous studies [1,4]. Similarly, lower BMI may reflect the nutritional consequences of prolonged dysphagia and impaired esophageal emptying, particularly among patients with achalasia. Although age-related changes in esophageal motor function have been described, older age in our study may reflect the likelihood of clinically significant motility disorders among patients undergoing specialist evaluation, although this association cannot distinguish disease-related effects from physiological aging. Referral indication also emerged as the strongest independent factor associated with the outcome, highlighting the importance of considering the clinical indication when interpreting HREM findings. However, given the retrospective referral-based design of the study, these associations should not be interpreted as evidence that referral-based patient selection improves diagnostic yield.

These findings have important implications for clinical practice in Mongolia. HREM is a specialized investigation with limited availability and currently remains centralized at a single institution. Consequently, understanding referral patterns and clinical characteristics associated with major esophageal motility disorders may help inform appropriate use of HREM in this setting. Our results suggest that patients presenting with dysphagia, unexplained esophageal symptoms, lower BMI, or clinical suspicion of a primary motility disorder were more likely to have major esophageal motility disorders than patients referred solely for reflux evaluation. These findings may help inform clinical assessment and referral decisions; however, the present study does not establish that such an approach improves diagnostic yield or reduces unnecessary investigations. Taken together, these findings support consideration of an indication-based approach to HREM referral, while prospective studies are needed to determine whether clinical selection criteria can improve diagnostic yield or optimize resource utilization.

The present study also provides important baseline data on HREM referrals and esophageal motility disorders for future development of esophageal motility services in Mongolia. As access to HREM expands nationally, these findings can serve as reference data for evaluating changes in referral practices, diagnostic yield, and disease patterns over time. Furthermore, standardized implementation of the Chicago Classification version 4.0 may facilitate more consistent diagnosis across centers and improve comparability with international studies [6,7,8].

Several strengths of this study should be acknowledged. To our knowledge, this is the first study to comprehensively evaluate HREM findings in Mongolia and includes a large HREM cohort from a low- and middle-income country. Consecutive patient inclusion reduced potential selection bias, standardized interpretation according to the Chicago Classification version 4.0 improved diagnostic consistency, and multivariable analyses enabled identification of clinical factors independently associated with major motility disorders. The sensitivity analysis excluding manometric EGJOO pattern further supported the consistency of the primary findings.

This study also has several limitations. First, the retrospective design limited data collection to routinely recorded clinical information and may have introduced information bias. Second, the study was conducted at a single tertiary referral center, although this institution currently provides the only routine clinical HREM service in Mongolia. Consequently, referral patterns may not fully represent the broader population of patients with esophageal symptoms, limiting the generalizability of the findings to other clinical settings. Third, symptom severity, quality-of-life measures, endoscopic findings, supportive investigations for EGJOO, and long-term clinical outcomes were not consistently available and therefore could not be evaluated. In particular, anatomical hiatal hernia was not systematically assessed by endoscopy or radiographic imaging. Although manometric EGJ morphology was available for all patients, LES–crural diaphragm separation on HREM should not be considered equivalent to an anatomically confirmed hiatal hernia. Furthermore, the absence of systematically available supportive investigations limited our ability to distinguish clinically relevant or conclusive EGJOO from manometric EGJOO patterns. Finally, because this was an observational study, causal relationships between clinical characteristics and major motility disorders cannot be established.

The multivariable analysis should also be interpreted cautiously. Although the model demonstrated moderate discrimination, its low sensitivity and suboptimal calibration limit its clinical utility. Moreover, the high overall classification accuracy was influenced by the low prevalence of major manometric motility abnormalities. The analysis was exploratory and was neither developed nor externally validated as a clinical prediction or screening tool. Accordingly, the identified associations should not currently be used to determine which patients should undergo HREM.

## 5. Conclusions

This study provides the first comprehensive characterization of high-resolution esophageal manometry findings among referred patients in Mongolia. Most examinations demonstrated either normal esophageal motility or ineffective esophageal motility, whereas major motility abnormalities were relatively uncommon. Older age, lower body mass index, dysphagia, and referral for suspected esophageal motility disorders were independently associated with major motility abnormalities. These findings provide baseline data for HREM practice in Mongolia. Given the retrospective single-center design and the exploratory nature of the multivariable analysis, these associations should not currently be used as a clinical prediction or screening tool. Prospective multicenter studies are needed to validate these findings and to further evaluate evidence-based HREM referral strategies.

## Figures and Tables

**Figure 1:**
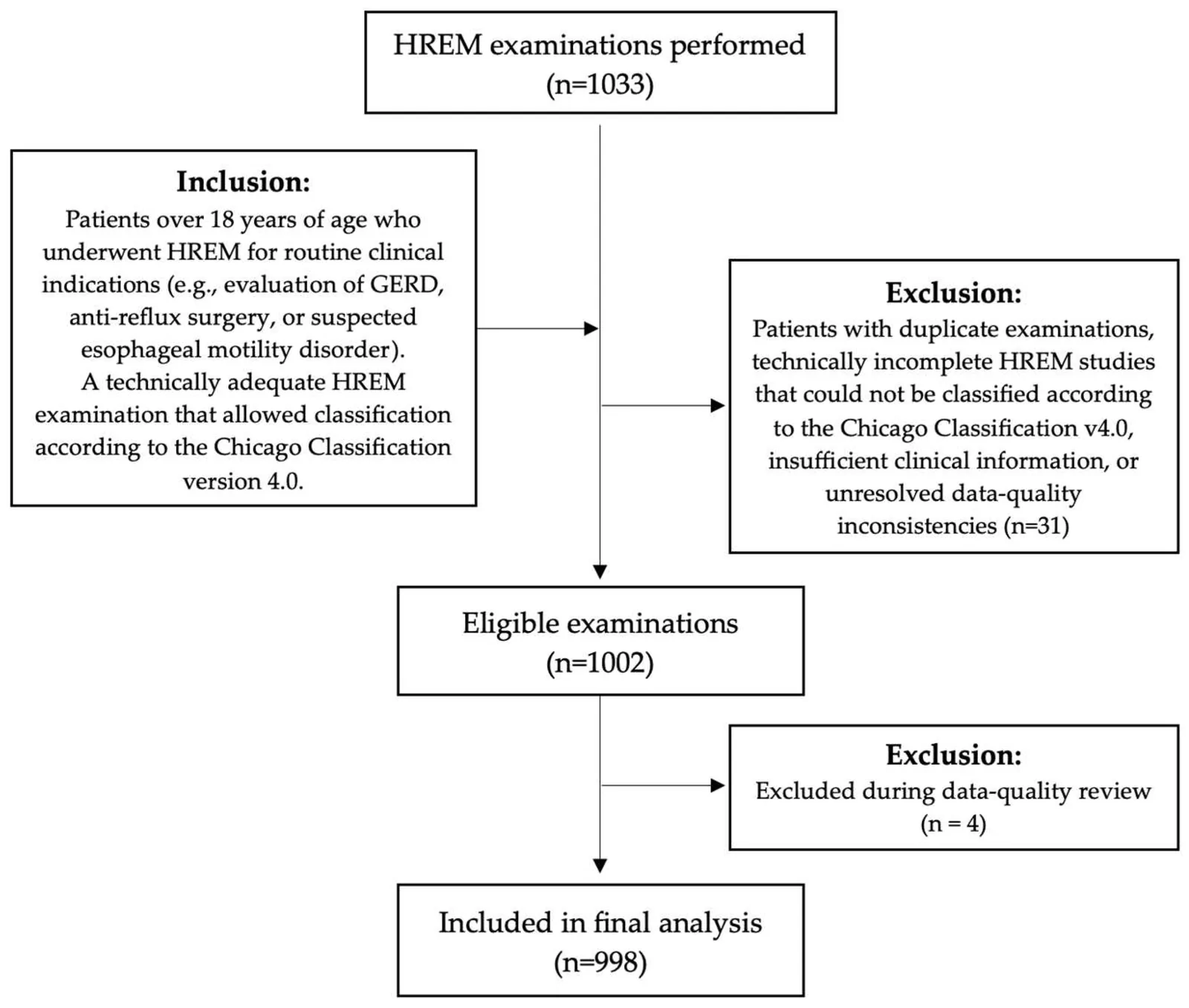
Study flow diagram.

**Figure 2:**
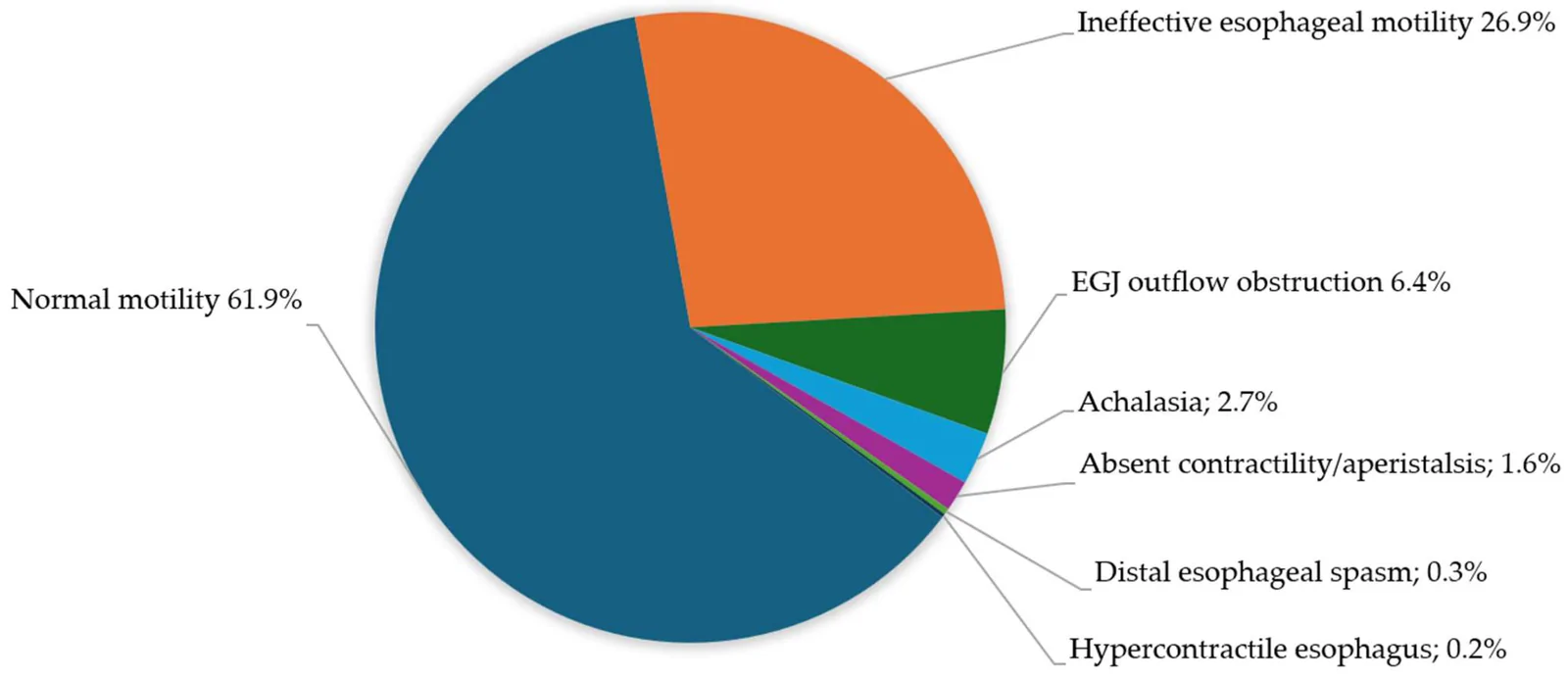
Distribution of esophageal motility disorders according to the Chicago Classification version 4.0.

**Figure 3:**
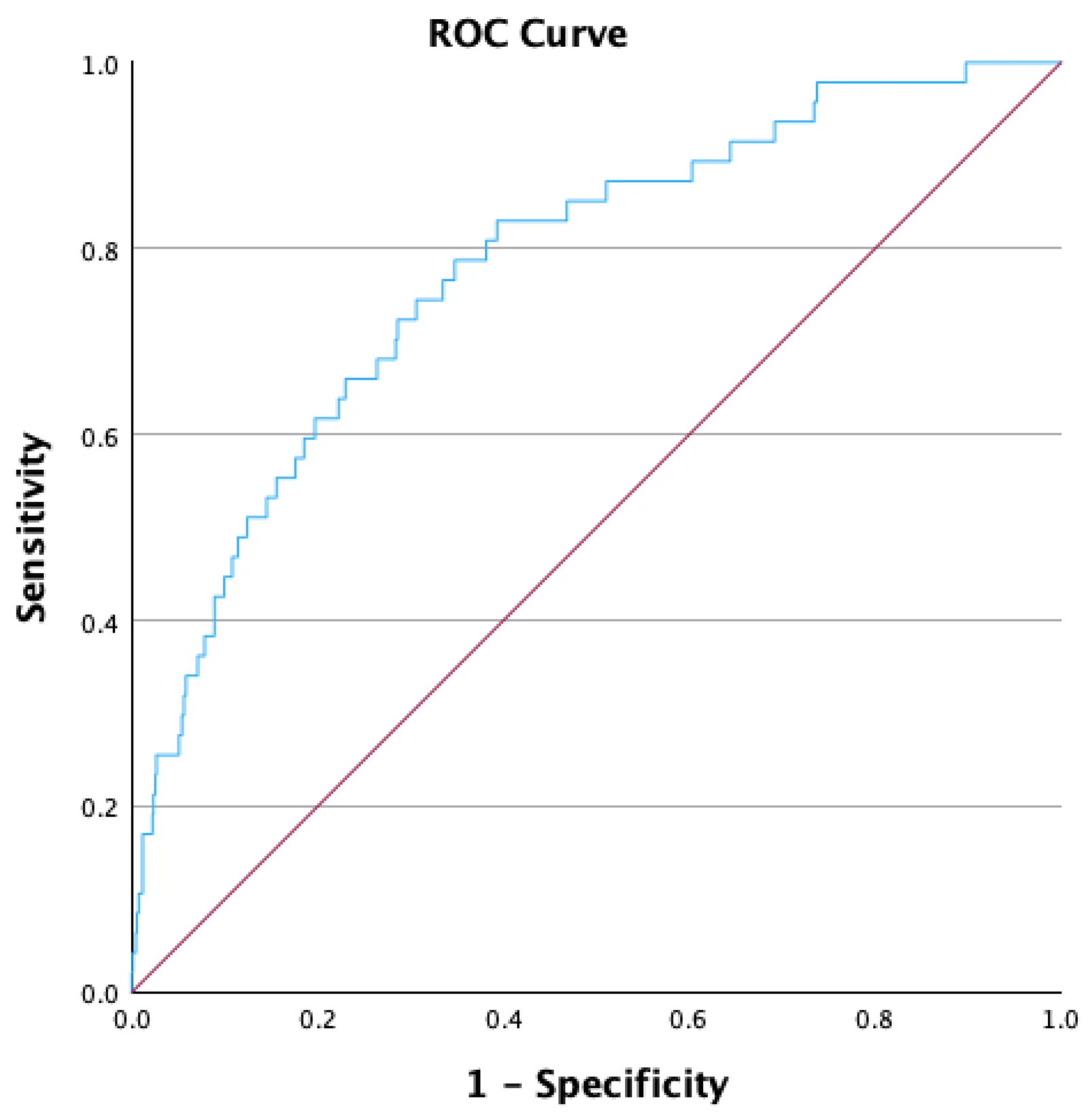
Receiver operating characteristic (ROC) curve of the exploratory multivariable logistic regression model for major manometric motility abnormalities. The blue line represents the ROC curve of the model, and the diagonal reference line represents chance-level discrimination (AUC = 0.50).

**Table 1:** Clinical characteristics according to referral indication.

Characteristic	Suspected GERD/Preoperative Anti-Reflux Assessment	Suspected Motility Disorder	*p* Value
Number of patients	696	302	-
Age, years	53.7 ± 12.5	48.9 ± 12.6	<0.001
Male sex	341/696 (49.0%)	85/302 (28.1%)	<0.001
BMI, kg/m^2^	27.9 ± 4.0	25.5 ± 4.7	<0.001
Number of symptoms	1.92 ± 0.91	1.70 ± 0.91	<0.001
Heartburn	636/691 (92.0%)	142/300 (47.3%)	<0.001
Regurgitation	276/691 (39.9%)	46/300 (15.3%)	<0.001
Dysphagia	92/691 (13.3%)	151/300 (50.3%)	<0.001
Non-cardiac chest pain	39/691 (5.6%)	67/300 (22.3%)	<0.001
Cough	151/691 (21.9%)	26/300 (8.7%)	<0.001
Belching	125/691 (18.1%)	64/300 (21.3%)	0.232

Data are presented as mean ± standard deviation (SD) or n (%). Percentages were calculated using the number of patients with available data for each variable. BMI, body mass index; GERD, gastroesophageal reflux disease.

**Table 2:** High-resolution esophageal manometry measurements according to referral indication.

HREM Measurement	Suspected GERD/Preoperative Anti-Reflux Assessment	Suspected Motility Disorder	*p* Value
Median IRP (mmHg)	9.0 (6.0–12.0)	14.0 (9.8–19.0)	<0.001
Mean DCI (mmHg∙sec∙cm)	702 (351–1176)	929 (457–1748)	<0.001
LES basal pressure (mmHg)	10 (5–17)	19 (10–26)	<0.001
LES length (cm)	3.7 (2.2–4.8)	2.9 (2.2–3.8)	<0.001
DCI ratio	1.21 (0.67–1.94)	1.16 (0.63–1.75)	0.222
Post MRS DCI (mmHg∙sec∙cm)	847 (302–1582)	1075 (350–2006)	0.005
Complete bolus transit, %	70 (50–80)	70 (40–80)	0.789

Data are presented as median (interquartile range, IQR). *p* values were calculated using the Mann–Whitney U test. DCI, distal contractile integral; IRP, integrated relaxation pressure; LES, lower esophageal sphincter; MRS, multiple rapid swallows.

**Table 3:** Characteristics according to major esophageal motility disorder status.

Characteristic	Normal/Minor Disorder	Major Disorders Including EGJOO	*p* Value
Number of patients	886	112	-
Age, years	52.2 ± 12.6	52.9 ± 13.7	0.586
Male sex	386/886 (43.6%)	40/112 (35.7%)	0.113
BMI, kg/m^2^	27.5 ± 4.2	24.7 ± 4.9	<0.001
Number of symptoms	1.86 ± 0.91	1.79 ± 0.99	0.465
Heartburn	717/880 (81.5%)	61/111 (55.0%)	<0.001
Regurgitation	296/880 (33.6%)	26/111 (23.4%)	0.030
Dysphagia	187/880 (21.3%)	56/111 (50.5%)	<0.001
Non-cardiac chest pain	91/880 (10.3%)	15/111 (13.5%)	0.308
Cough	164/880 (18.6%)	13/111 (11.7%)	0.073
Belching	168/880 (19.1%)	21/111 (18.9%)	0.965
Suspected motility-disorder referral	223/886 (25.2%)	79/112 (70.5%)	<0.001

Data are presented as mean ± standard deviation (SD) or n (%). Percentages were calculated using the number of patients with available data for each variable. EGJOO, esophagogastric junction outflow obstruction; BMI, body mass index.

**Table 4:** Logistic regression analysis of factors associated with major manometric motility abnormalities.

Variable	Unadjusted OR (95% CI)	*p* Value	Adjusted OR (95% CI)	*p* Value
Age, per 10 years	1.04 (0.89–1.22)	0.586	1.35 (1.12–1.61)	0.001
Male sex	0.72 (0.48–1.08)	0.115	1.41 (0.88–2.25)	0.155
BMI, per kg/m^2^	0.85 (0.80–0.89)	<0.001	0.90 (0.85–0.95)	<0.001
Heartburn	0.28 (0.18–0.42)	<0.001	0.85 (0.51–1.41)	0.530
Regurgitation	0.60 (0.38–0.96)	0.032	-	-
Dysphagia	3.77 (2.52–5.66)	<0.001	1.95 (1.20–3.15)	0.007
Non-cardiac chest pain	1.36 (0.75–2.43)	0.310	-	-
Cough	0.58 (0.32–1.06)	0.076	-	-
Belching	0.99 (0.60–1.64)	0.965	-	-
Suspected motility-disorder referral	7.12 (4.61–10.98)	<0.001	5.47 (3.22–9.31)	<0.001

OR, odds ratio; CI, confidence interval; BMI, body mass index. Adjusted odds ratios were obtained from the multivariable logistic regression model including age, sex, body mass index, dysphagia, heartburn, and referral indication.

**Table 5:** Sensitivity analysis of factors associated with major motility abnormalities after excluding manometric EGJOO.

Predictor	Adjusted OR (95% CI)	*p* Value
Age (per 10 years)	1.30 (1.02–1.65)	0.037
Male sex	2.19 (1.15–4.17)	0.017
BMI	0.90 (0.83–0.97)	0.009
Heartburn	0.91 (0.44–1.88)	0.799
Dysphagia	2.46 (1.23–4.90)	0.011
Suspected motility disorder referral	3.79 (1.75–8.21)	<0.001

OR, odds ratio; CI, confidence interval; BMI, body mass index; EGJOO, esophagogastric junction outflow obstruction. The outcome was redefined after excluding esophagogastric junction outflow obstruction from the major esophageal motility disorder group.

## Data Availability

The data used to support the findings of this study are available from the corresponding author upon request.
